# Multiple-population QTL mapping of maturity and fruit-quality traits reveals LG4 region as a breeding target in sweet cherry (*Prunus avium* L.)

**DOI:** 10.1038/s41438-020-00349-2

**Published:** 2020-08-01

**Authors:** Alejandro Calle, Ana Wünsch

**Affiliations:** 1 Unidad de Hortofruticultura, Centro de Investigación y Tecnología Agroalimentaria de Aragón (CITA). Avda. Montañana 930, 50059 Zaragoza, Spain; 2 Instituto Agroalimentario de Aragón-IA2 (CITA-Universidad de Zaragoza), Zaragoza, Spain

**Keywords:** Plant breeding, Plant breeding

## Abstract

Sweet cherry maturity date and fruit quality are relevant traits for its marketability, transport, and consumer acceptance. In this work, sweet cherry fruit development time, maturity date, and commercial fruit-quality traits (size, weight, firmness, soluble solid content, and titratable acidity) were investigated to improve the knowledge of their genetic control, and to identify alleles of breeding interest. Six sweet cherry populations segregating for these traits were used for QTL analyses. These populations descend from cross- and self-pollinations of local Spanish sweet cherries ‘Ambrunés’ and ‘Cristobalina’, and breed cultivars (‘Brooks’, ‘Lambert’, or ‘Vic’). The six populations (*n* = 411), previously genotyped with RosBREED Cherry 6 K SNP array, were phenotyped for 2 years. QTL analyses were conducted using a multifamily approach implemented by FlexQTL^™^. Fruit development time, soluble solid content, and titratable acidity QTLs are first reported in sweet cherry in this work. Significant QTLs were detected for all the traits. Eighteen were more stable as they were detected for 2 years. Of these, nine are first reported in this work. The major QTLs for fruit development time, maturity date, firmness, and soluble solid content were identified on the same narrow region of linkage group 4. These traits also showed significant positive correlation (long fruit development time associated with late maturity, high firmness, and high SSC). *NAC* transcription factor genes identified on this LG4 region may be candidate genes for the regulation of these traits in sweet cherry, as previously described in syntenic regions of other Rosaceae species. Haplotypes of breeding interest on this LG4 genomic region were identified and will be useful for sweet cherry breeding from this and related plant material.

## Introduction

Sweet cherry (*Prunus avium* L.) is a highly demanded fruit as it is the earliest stone fruit to ripen and because of its great consumer acceptance. Sweet cherries are mainly consumed as fresh fruit, and therefore, their market profitability is directly related to their maturity date (MD) and fruit quality^1^. As early ripening cultivars generally command the highest prices^2^, understanding the genetics of MD is highly relevant for breeding. Late-maturing cultivars are also a breeding target, as these allow extending the harvesting season^3^. Sweet cherry MD is related to bloom date and fruit development (FD), both of which vary between cultivars. In apricot and peach, it has been observed that FD and MD also display broad variability and a large positive correlation^4,5^. Early and late blooming are also breeding objectives for market and crop adaptation^6^. In colder regions, late blooming may be desired to avoid damage due to spring freezes, and in warmer regions, low chilling requirement cultivars are desired to ensure flowering. Thus, the investigation of FD time and MD, their relationship, and that with bloom time (BT) is of high interest for breeding for early and late maturity in sweet cherry and cultivar adaptation to different growing areas.

Quantitative trait locus (QTL) analyses have been conducted in sweet cherry to investigate the genetic control of MD^7–9^ but not FD period. Analysis of MD in a ‘Regina’ × ‘Lapins’ population during 3 years^7,8^ identified three stable QTLs on linkage groups (LGs) 1, 4, and 5. A large percentage of variation was explained by a QTL on LG4 (20.4%), which was associated with advancing maturity 5.4 days^7^. This major MD QTL was also identified by Isuzugawa et al.^9^ in another segregating sweet cherry population (‘Beniyutaka’ × ‘Benikirari’). In this case, LG4 QTL explained 48.4% of the variation. The same LG4 QTL has been reported in other *Prunus* species, including apricot (*Prunus armeniaca* L.), peach (*Prunus persica* L.), and plum (*Prunus salicina* Lindl.) as the main MD QTL^5,7,10–12^. FD time has also been investigated in *Prunus*, but only in apricot and peach. A main QTL for FD time was found on the same LG4 genomic region where the MD QTL has been identified in these species^5,13,14^. In this region, *NAC* transcription factors have been reported as candidate genes for MD in peach^15^ and other *Rosaceae* species like apple^16^.

The other main attribute for sweet cherry marketability is fruit quality. Fruit quality depends on various fruit characteristics, including size, taste, flavor, firmness, sweetness, acidity, color, and external appearance^1,2,17^. Consumer preferences regard large size, sweetness, firmness, long shelf life, and adequate balance between soluble solid content (SSC) and titratable acidity (TA) as the main attributes to select sweet cherries^18,19^. Various fruit-size (FS) QTLs have been published in sweet cherry^20–23^. Zhang et al.^20^ reported FS and weight QTLs on LGs 2 and 6 of the population ‘New York 54’ × ‘Emperor Francis’. Rosyara et al.^21^ detected four QTLs associated with fruit weight (FW) on LGs 1–3, and Campoy et al.^22^ reported a major FW QTL at the bottom region of ‘Regina’ LG5. QTLs and alleles of breeding interest for sweet cherry FS have been identified and validated on LG1 of the Spanish landrace ‘Ambrunés’^23^. FF in sweet cherry has also been investigated in various works^22–24^. Two major QTLs on LGs 2 and 5 and various minor QTLs were initially reported^22^. A more recent study using a larger sample of cultivars and populations revealed a major QTL on LG4, explaining up to 84.6% of FF variation^24^. In addition, Calle et al.^23^ have validated firmness QTLs on LGs 1 and 6 in a different genetic background, and identified alleles of breeding interest on LG1. QTL analyses of fruit acidity and soluble solid content have been reported in apricot, peach, and plum^10,12,25–27^, but not in sweet cherry. QTLs controlling these traits in these species were found on apricot LGs 1, 2, 4, and 5^27^, on peach LGs 4 and 5^10,25,26^, and on plum LGs 1 and 6^12^.

The use of single biparental populations for QTL analyses and mapping strategies limits QTL detection and the potential of results for marker-assisted selection in a wide range of plant materials with different genetic backgrounds. Only three studies on FS^21^, FF^24^, and BT^6^ have combined a large number of individuals from multiple sweet cherry populations for QTL analysis. These works have resulted in the identification of large stable QTLs for these traits. Thus, larger studies are necessary to understand maturity and fruit-quality genetics in sweet cherry. With this aim, in this work, a multifamily approach was used to identify QTLs associated with sweet cherry FD time, MD, and fruit-quality traits. QTLs for some of these traits (FD time, SSC, and TA) are first investigated for this species herein. In addition, this multifamily approach was carried out using four F_1_ and two F_2_ sweet cherry populations that descend from Spanish local plant material of breeding interest (‘Ambrunés’ and ‘Cristobalina’)^6,23^ and breed cultivars. These materials present a wide phenotypic range for these traits and combine distant genetic pools. The same plant materials, which include the only two F_2_ populations reported in sweet cherry for genetic analysis, were previously successfully used to investigate BT genetics using the same approach^6^.

## Results

### Phenotyping

All data recorded were used for the analyses as no outlier data were detected. Phenotyping of parental cultivars revealed differences for all traits between years and between cultivars (Supplementary Table 1a). Differences between cultivars were the largest for FD time and MD. For FD, differences of almost 3 weeks (17–20 days per year) were observed between the cultivar with the largest and shortest FD time. For MD, differences of more than 7 weeks (35–41 days per year) were recorded between the earliest and latest parental cultivar to mature. Cultivars ‘Ambrunés’, ‘Lambert’, and ‘Vic’ showed the largest FD (around 11 weeks) and the latest MD (June 2nd to 22nd). On the other hand, ‘Cristobalina’ and ‘Brooks’ exhibited shorter FD (8–9 weeks) with ‘Cristobalina’ showing the earliest MD (May 2nd to 18th, Supplementary Table 1a).

For fruit-quality traits, significant differences were observed for those traits with means comparison: FW, FS, and FF. Fruit-quality traits with the largest differences between parental cultivars were FW (5–6 g per year), FS (6–8 mm each year), and FF (17–22% per year, Supplementary Table 1a). The largest cherries were collected from ‘Ambrunés’, ‘Brooks’, ‘Lambert’, and ‘Vic’ with values of 8–9 g and 25–28 mm. ‘Cristobalina’ was the cultivar with the smallest fruits, which were 4 g and 19–20 mm (Supplementary Table 1a). For FF, ‘Lambert’ showed lower values both years (33–47%), while ‘Ambrunés’, ‘Brooks’, and ‘Cristobalina’ were firmer both years (49–63%, Supplementary Table 1a). For SSC and TA, parental values showed smaller variation between years and cultivars, and the results did not reveal any pattern for the same cultivars among years (Supplementary Table 1a).

**Fig. 1:**
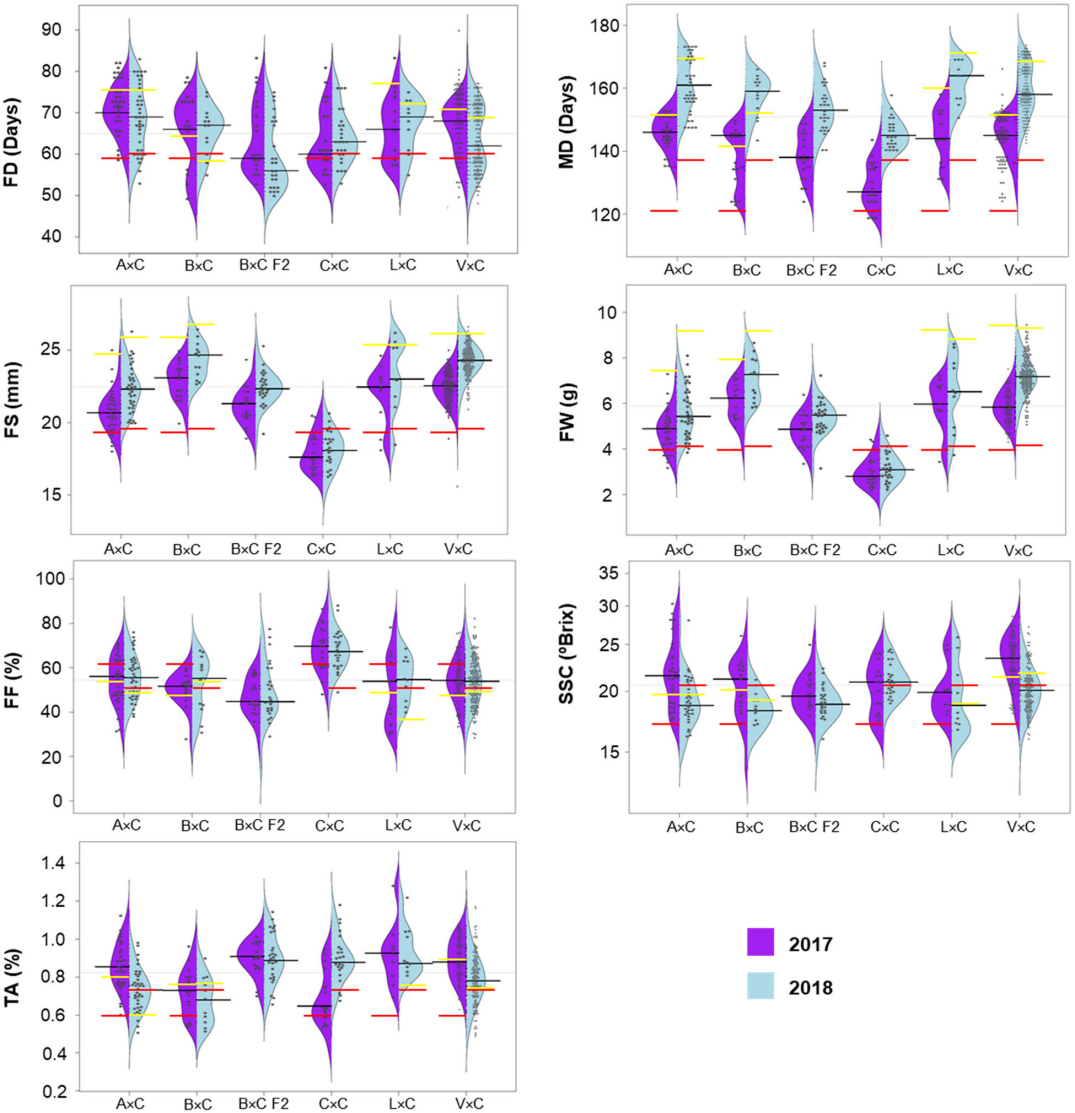
Violin-plot distribution and phenotype individual values (dots) of fruit development time (FD), maturity date (MD), fruit size (FS), fruit weight (FW), fruit firmness (FF), soluble solid content (SSC), and titratable acidity (TA) per population, in years 2017 (purple) and 2018 (blue). Black lines indicate median values. Red lines indicate ‘Cristobalina’ phenotypic values (female parental values in F_1_s) and yellow lines indicate male parental values

In the six populations, 197 trees (48% of genotyped trees) were phenotyped in 2017 and 257 (63%) in 2018 (Supplementary Table 1b). These data revealed variability in all the populations for all the traits (Fig. 1). Significant differences between years for mean phenotype values were observed for all traits, except for FD and FF (Student’s *t* test, *p* < 0.05 Fig. 1; Supplementary Table 1b). For all the populations, fruits were larger, heavier, and matured later in 2018 than in 2017, except in C×C, in which sweeter and less-acid fruits were harvested in 2017 (Fig. 1; Supplementary Table 1).

Phenotypic segregation for all traits was observed in the six populations both years (Fig. 1; Supplementary Table 1b). For FD, differences larger than a month (36 days, more than 5 weeks) between the shortest (48 days, nearly 7 weeks, trees B×C F2-51 and V×C-104, 2018) and the largest (84 days, 12 weeks, tree V×C-26, 2017) FD period were observed (Fig. 1; Supplementary Table 1b). Progeny from B×C F2 exhibited the shortest FD, while A×C and L×C progenies exhibited the widest FD (Fig. 1). MD segregation ranged from 5 to 6 weeks each year (120–163 days in 2017 and 138–173 days in 2018) with MDs ranging from early May to late June in both years (Fig. 1; Supplementary Table 1b). Progeny from the C×C population, followed by B×C F2, showed the earliest MD ripening on average 16 days earlier than individuals from the latest populations (A×C and L×C, Fig. 1; Supplementary Table 1b).

Large variation was also observed for FS and FW within populations. FS population means ranged from 5 g/7 mm to 8 g/11 mm both years (Fig. 1; Supplementary Table 1). The smallest cherries were those of C×C, whereas F_1_ populations from bred cultivars (B×C and V×C) had the largest cherries (Fig. 1). FF displayed broad variation in the populations with values ranging from 15 to 87% both years (Supplementary Table 1). Similar FF was observed for A×C, B×C, L×C, and V×C means (50–56%). The firmest fruits were identified in C×C (68% both years) and the softest in B×C F2 (46–48%) (Fig. 1; Supplementary Table 1b). For SSC and TA, large variability was observed both years in all the populations, and generally values in 2018 were lower than in 2017 (Fig. 1; Supplementary Table 1). The exception was C×C, in which TA mean value was larger in 2017 than in 2018 (Fig. 1; Supplementary Table 1). Individual progeny means varied from 13 to 30 °Brix for SSC and from 0.43 to 1.28% for TA. Mean TA was the highest in B×C F2 and L×C, while SSC was the highest in A×C and V×C (Fig. 1; Supplementary Table 1b).

**Fig. 2:**
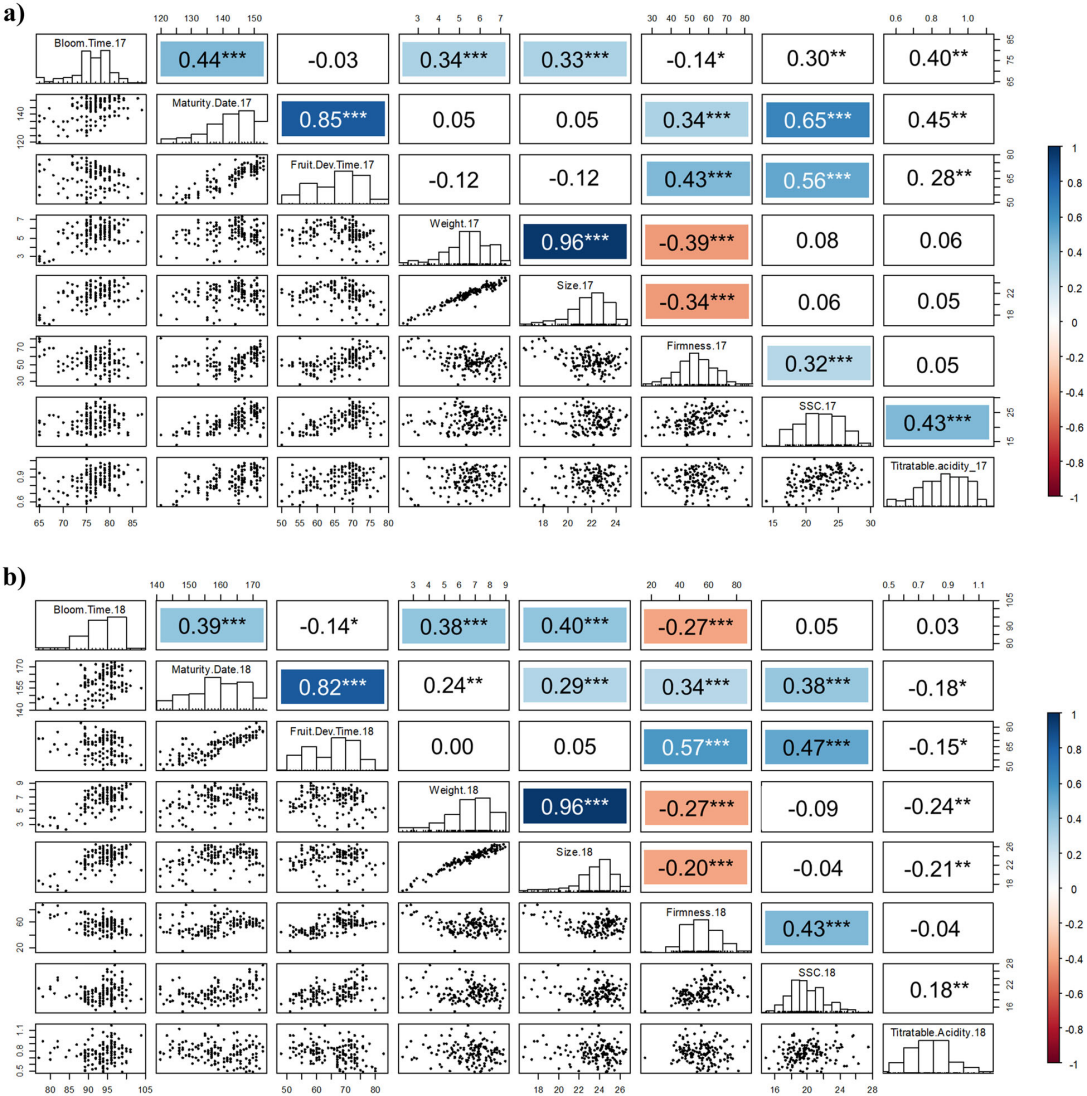
Spearman correlation coefficient among traits, trait distribution histograms, and correlation plots in 2017 (**a**) and 2018 (**b**). Asterisks indicate correlation significance (*p < 0.01; **p < 0.001; ***p < 0.0001). Positive and negative correlations at *p* < 0.0001 are marked in blue and orange, respectively

Trait distributions were similar both years (Fig. 2). Only FF, SSC, and TA fitted normal distributions, whereas the remaining traits (FD, MD, FW, and FS) exhibited skewed distributions toward longer FD, late MD, and large fruit (Shapiro–Wilk test). A bimodal distribution for FD was observed both years (Fig. 2).

Values of broad-sense heritabilities (*H*^2^) calculated for both years varied among traits and ranged from 0.54 for TA to 0.94 for MD (Table 1). Large broad-sense heritability was also observed for FD (0.92), FS (0.93), FW (0.92), and FF (0.84), while a lower *H*^2^ was detected for SSC (0.62) (Table 1).

**Table 1:** QTLs detected both years

	*H*^2^	QTL name	LG	Genetic position (cM)	Physical position* (Mbp)	Average 2lnBF (2017/2018)	Additive effect (2017/2018)	PVE (%) (2017/2018)
Fruit development time (FD)	0.92	*qP-FD3.1*^*m*^	3	17–62	4.16–19.65	3.4/6.2	2.8/3.1	2.7/6.9
		*qP-FD4.1*^*m*^	4	8–32	1.98–6.82	3.9/5.9	5.1/5.7	5.8/18.2
		**qP-FD4.2**^***m***^	4	51–53	10.88–11.66	11.7/11.8	10.8/11.7	65.3/64.5
Maturity date (MD)	0.94	*qP-MD1.1*^*m*^	1	50–77	14.33–28.94	3.8/7.6	3.4/4.1	5.4/8.6
		**qP-MD2.1**^***m***^	2	68–76	25.24–29.94	6.1/11.7	3.9/4.9	11.7/10.4
		*qP-MD3.1*^*m*^	3	13–52	3.70–15.84	3.5/8.1	5.4/3.8	19.5/6.7
		*qP-MD4.1*^*m*^	4	5–33	1.98–6.82	2.7/7.7	3.9/6.1	4.2/11.0
		**qP-MD4.2**^***m***^	4	51–53	10.88–11.66	9.5/11.8	11.1/11.6	46.8/52.5
		*qP-MD5.1*^*m*^	5	57–71	13.62–18.41	4.7/8.1	2.2/2.3	2.1/2.8
Fruit weight (FW)	0.92	*qP-FW1.1*^*m*^	1	42–84	11.08–30.61	5.2/4.5	0.8/1.1	6.1/15.7
		*qP-FW2.1*^*m*^	2	31–74	17.86–28.60	4.9/5.9	1.0/1.7	23.9/53.9
		*qP-FW5.1*^*m*^	5	31–54	8.42–13.18	6.5/4.4	1.5/1.3	45.4/6.9
Fruit size (FS)	0.93	**qP-FS2.1**^***m***^	2	57–76	23.74–29.94	6.7/7.2	1.4/1.1	23.6/21.5
Fruit firmness (FF)	0.84	**qP-FF4.1**^***m***^	4	50–54	10.41–12.57	11.7/9.5	14.4/15.0	47.9/64.1
		*qP-FF6.1*^*m*^	6	74–109	22.65–30.45	4.8/2.3	3.7/2.9	2.5/1.3
Soluble solid content (SSC)	0.62	*qP-SSC3.1*^*m*^	3	18–69	4.50–21.85	4.8/4.2	1.5/0.9	10.4/7.4
		**qP-SSC4.1**^***m***^	4	50–59	10.41–13.10	11.7/6.8	3.0/1.7	34.2/22.1
Titratable acidity (TA)	0.54	**qP-TA6.1**^***m***^	6	91–108	26.77–30.45	9.6/6.3	0.09/0.07	21.6/15.0

Genetic and physical positions spanned both years, average Bayes Factor (*2lnBF*), mean additive effect, and percentage of variance explained (PVE) both years. In bold, QTLs with decisive and/or strong evidence (*2lnBF* > 5) both years. A full list of all detected QTLs, including those only identified in a single year, is found in Supplementary Table 2

### Trait correlations

Correlations between traits were similar both years (Fig. 2; Supplementary Table 1). A high significant positive correlation was observed between FD and MD (0.82–0.85), and between FW and FS (0.96). These results revealed that fruits that took longer to develop were also the latest to mature, and that the largest fruits were also heavier. FD and MD also showed moderate significant positive correlations with SCC (0.65/0.56 in 2017, and 0.36/0.47 in 2018, respectively). In addition, FD was positively and significantly correlated with FF (0.43 in 2017, and 0.57 in 2018) (Fig. 2). That is, latter cherries and those that took longer to develop tended to be firmer and sweeter (Fig. 2).

Correlation analysis of BT data reported previously for the same plant material and the same years^6^, revealed significant moderate positive correlation with MD (0.44 and 0.39 in 2017 and 2018, respectively), FW (0.34 and 0.38 in 2017 and 2018, respectively), and FS (0.33 and 0.40 in 2017 and 2018, respectively, Fig. 2). A negative low–moderate correlation was also observed between FF and FS, and FW (from −0.20 to −0.39), indicating that larger fruits tended to be softer (Fig. 2).

### QTL analysis

**Fig. 3:**
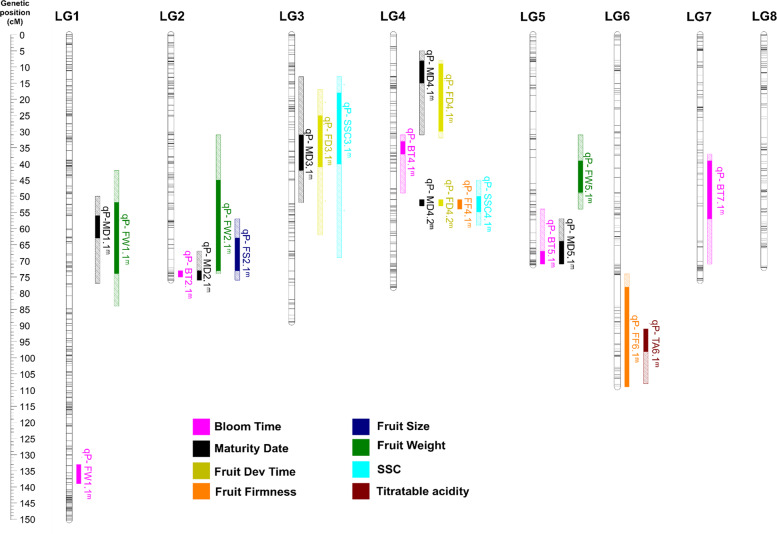
Genetic positions on the consensus linkage map^48^ of QTLs detected both years. QTL interval overlapping both years is shown in bold, intervals detected only 1 year are shown with diagonal bars. All QTLs detected, including each year interval, are shown in Supplementary Fig. 1. Bloom-time QTLs shown were previously published^6^ and correspond to data from the same plant material and same years

QTLs were detected for all traits both years (Supplementary Table 2; Supplementary Fig. 1). A total of 30 QTLs were detected for the seven traits (5 for FD, 6 for MD, 6 for FW, 5 for FS, 2 for FF, 3 for SSC, and 3 for TA; Supplementary Table 2; Supplementary Fig. 1). Eighteen of these were considered stable as they were detected both years, being three for FD, six for MD, three for FW, one for FS, two for FF, two for SSC, and one for TA (Table 1, Fig. 3). Of these stable QTLs, one FD QTL was detected with decisive evidence (*2lnBF* > 10) both years, and another six were detected with strong evidence (*2lnBF* > 5) both years (two for MD, one for FS, one for FF, one for SSC, and one for TA, Table 1; Fig. 3). Another 12 QTLs (2 for FD, 2 for FW, 5 for FS, 1 for SSC, and 2 for TA) were detected only 1 year (Supplementary Table 2; Supplementary Fig. 1).

The proportion of phenotype variance explained (PVE) by each QTL ranged from 0.4 to 65.3% (Supplementary Table 2). QTLs detected for FD, MD, FW, and FF explained more than 60% of total phenotypic variation. QTLs detected for SSC and TA explained between 20 and 50% of the total phenotypic variation (Supplementary Table 2).

QTLs were detected on LGs 1–6 (Supplementary Fig. 1). Three stable and significant QTLs for FD were detected on LGs 3 and 4 (Table 1, Fig. 3). The most significant, *qP-FD4.2*^*m*^, is found in a narrow region (51–53 cM) of LG4, explained a large portion of the variation (PVE: 65.3–64.5%), and had an additive effect of 11–12 days (Table 1, Fig. 3). For MD, six stable QTLs were found, two of them detected with decisive evidences. One of them, *qP-MD4.2*^*m*^, is on the same LG4 region where the major FD QTL (*qP-FD4.2*^*m*^) was identified. This result is consistent with the high correlation between these two traits. This MD QTL on LG4 showed the largest PVE (46.8–52.5%) and similar additive effects to the FD QTL, 11–12 days each year (Table 1, Fig. 3). The other QTL with decisive evidence for MD was on LG2 (*qP-MD2.1*^*m*^), but explained a lower percentage of variation (10.4–11.75%, Table 1).

*NAC* transcription factors have been described as candidate genes for MD in *Prunus*^11,15^. In this work, *NAC* transcription factors were searched for in the sweet cherry genome (PAV_r1.0)^28^ for colocalization with the major FD and MD QTLs on LG4 (*qP-FD4.2*^*m*^ and *qP-MD4.2*^*m*^). This QTL region (LG4: 51–53 cM) corresponds with PAV_r1.0 physical region 13,304,990–14,860,789 bps in chromosome 4. Two genes annotated as *NAC* transcription factors, *Pav_sc0000029.1_g070.1.mk* and *Pav_sc0000029.1_g090.1.mk*, were identified in this physical region. These two genes are 19.2 kb away from each other and are flanked by SNP markers ss490552906 and ss490552928.

For FW, three stable QTLs (*qP-FW1.1*^*m*^, *qP-FW2.1*^*m*^, and *qP-FW5.1*^*m*^) were identified although none of them were detected with decisive evidence (Table 1, Fig. 3). The PVE explained by these three QTLs varied largely between years, with the largest effects observed for *qP-FW2.1*^*m*^ and *qP-FW5.1*^*m*^ in 2017 and 2018, respectively (Table 1). For FS, a stable QTL was identified on LG2 (*qP-FS2.1*^*m*^; PVE 21.5–23.6) in the same region as FW QTL *qP-FW2.1*^*m*^ (Table 1, Fig. 3). Regarding FF, a major stable QTL was detected on LG4 (*qP-FF4.1*^*m*^) (Table 1, Fig. 3). This QTL that also showed decisive evidences both years, located to a narrow interval of 50–54 cM on LG4 (Table 1, Fig. 3). This corresponded to the same genomic region where the major QTLs for FD and MD were detected (Fig. 3). *qP-FF4.1*^*m*^ explained a large PVE (47.9–64.1%) and exhibited additive effects for FF of 14.4–15.0% (Table 1).

Regarding SSC, a major stable QTL was also found on the same region of LG4 in which the major FD, MD, and FF QTLs were detected (Table 1, Fig. 3). *qP-SSC4.1*^*m*^ was located between 50 and 59 cM of LG4 and explained the largest PVE (34.2–22.1%) with values varying between 1.7 and 3.0 °Brix (Table 1, Fig. 3). For TA, a stable QTL, *qP-TA6.1*^*m*^, was detected on LG6 explaining from 15.0 to 21.6% of PVE (Table 1, Fig. 3).

### Haplotype analysis of LG4

**Fig. 4:**
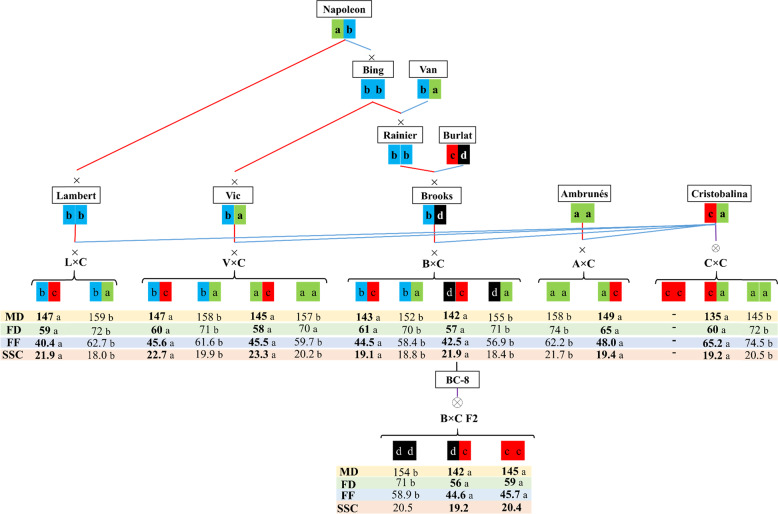
Haplotypes of LG4 major QTLs for fruit development period (FD), maturity date (MD), and firmness (FF) QTLs (*qP-FD4.2*^*m*^*/qP-MD4.2*^*m/*^*qP-FF4.1*^*m*^; LG4: 50–54 cM) in parental and ancestor cultivars, and in each population. Mean phenotype values of both years of each segregating class detected in each population are shown. Values for these haplotypes are also shown for SSC. Means significant differences between segregating classes are identified by different letters (*p* < 0.05)

Haplotype analysis of the LG4 region of 50–54 cM, spanning the stable and decisive QTLs for FD (*qP-FD4.2*^*m*^), MD (*qP-MD4.2*^*m*^), and FF (*qP-FF4.1*^*m*^) was carried out for parental cultivars and their ancestors (Fig. 4; Supplementary Table 3). Four haplotypes (*H4-a* to *-d*) were identified using the six SNPs that span this LG4 interval (10.41–11.66 Mbp) (Supplementary Table 3). *H4-a* and *H4-b* were the most frequent haplotypes being present in all parental and ancestor cultivars, except in ‘Burlat’ (Fig. 4; Supplementary Table 3). Haplotypes *H4-c* and *H4-d* were only found in ‘Burlat’ (*H4-c/H4-d*), ‘Cristobalina’ (*H4-c*), and in their descendants (‘Brooks’, *H4-d;* ‘BC-8’, *H4-c/H4-d*) (Fig. 4; Supplementary Table 3).

Comparison of mean phenotypic values for LG4 50–54 cM interval in the populations revealed that within families, individuals with haplotype *H4-c* had significantly shorter FD and earlier MD than individuals without it (Fig. 4; Supplementary Table 4). These individuals (genotypes *c-* and *cc*) developed and ripened on average 12 days earlier than individuals with other haplotype combinations (*aa, ab, ad, dd*) (Fig. 4; Supplementary Table 4). Individual differences for FD were as large as 5 weeks (36 days) for individuals with and without *H4-c*, whereas for MD, differences of 1 month were observed (Figs. 1 and 2). For these traits, also a dominant effect of *H4-c* was observed when compared with *H4-d* in B×C F2. In addition, within populations, those individuals with genotypes ‘*c-*’ or ‘*cc*’ showed significantly lower firmness than individuals with other haplotype combinations (Fig. 4; Supplementary Table 4). Consequently, within families, FF was higher in individuals with larger FD and later MD, which corresponded to individuals with the other haplotype combinations observed in this LG4 region, namely ‘*aa*’, ‘*ab*’, *‘ad’*, and ‘*dd’* (Fig. 4; Supplementary Table 4). No other significant differences were observed for the other diplotypes within families.

Using the same haplotypes (LG4: 50–54 cM), phenotypic value mean comparison of the segregating classes within populations was also carried out for SSC. The major SSC QTL detected in this work on LG4 (50–59 cM) is also overlapping with the LG4 region where the main FD, MD, and FF has been detected (LG4: 50–54 cM). In addition, a significant positive correlation between SSC and FD, MD, and FF has also been observed in this work (Fig. 2). Therefore, although the estimated haplotypes on LG4 do not span the complete SSC QTL region, it is relevant to explore their SSC phenotypic value in order to quantify the effect in SSC when selecting for these haplotypes (Fig. 4). As observed for FF within families, SSC was significantly lower in individuals with ‘*c-*’ or ‘*cc*’ than with other haplotype combinations. Therefore, individuals with larger FD and later MD had a higher SSC, and vice versa.

## Discussion

### BT, FD, and MD

Understanding and identifying trait correlations provide knowledge for a more efficient phenotyping and breeding selection. Low positive correlation or no correlation has been previously reported in sweet cherry for BT and MD^7,29,30^. In this study also, a moderate positive correlation between these two traits was observed. This correlation was most evident in the earlier blooming and ripening cultivars. The inclusion in this work of C×C, which shows extra-early blooming^6^ and MDs, may have biased the correlation of these two traits when analyzing all the plant material. The results, therefore, suggest that BT and MD are not completely correlated, and as observed in many cultivars, not all early-blooming genotypes are early maturing, or vice versa.

The high positive correlation between MD and FD time, observed herein for the first time in sweet cherry, has been previously observed in peach and apricot^4,5,27^. The results indicate that MD is highly dependent on FD time in sweet cherry, as has been previously observed in other *Prunus* species. However, a low positive correlation observed between BT and MD, also indicates that sometimes, or to a certain degree, MD also depends on BT. If blooming takes place earlier, as it happened in 2017, then maturity also takes place earlier, or vice versa, independently of FD time. BT and FD time are independent of each other, as no correlation between them was observed. However, MD is correlated to both, thus depends on both, but on FD time to a larger extent. In terms of breeding, this result translates to the possibility of combining BT and FD time phenotypes to achieve specific breeding goals for the MD. To either try to advance or delay MD by using short or long FD time, and to try to adapt to environmental conditions by introducing late (avoidance of late frosts) or early blooming (low chilling).

High *H*^2^ was estimated for most traits analyzed. As previously described in other *Prunus* species^31^, broad-sense heritability was the highest for FD time and MD. The *H*^2^ estimated for FD time (0.92) and MD (0.94) was similar to that estimated previously in sweet cherry (MD *H*^2^: 0.76–0.83, refs. ^7,30^) and in peach (FD time *H*^2^: 0.88–0.92, refs. ^13,14^). As FD time has not been evaluated previously in sweet cherry, the results herein confirm previous works in *Prunus* in which FD time also showed very high heritability^13,14^. Despite the large heritability, FD time showed small variability between years, while for MD, large differences were observed. On average, maturity reached 20 days earlier in 2017 than in 2018. The same year effect was observed in the BT of the same plant material the same years^6^, with blooming being 20 days earlier in 2017 than in 2018. Thus, as discussed above, in this work, interannual variation had a large effect in both MD and BT.

Traits showing normal (MD) and bimodal (FD time) distribution were observed in this study, revealing different behavior of these traits. As reported here, normal distribution was previously observed for MD in sweet cherry^8^ and apricot^5,27^. However, in most peach populations, bimodal distributions for MD were reported^10,11,25^. These populations include individuals of early and late ripening, which suggests the presence of a major locus governing the trait. The same type of bimodal distribution was observed in this work for FD time, but not for MD. This difference may be explained by the differences in FD time in these species. In cherries, FD time is shorter than in peach, and therefore, BT may have a larger effect on MD than it has in peach. In peach, FD time is much longer, and therefore the influence of BT on MD may be much smaller.

The interaction and correlation between BT, FD time, and MD was also evidenced by the QTL analysis in this work. The major QTLs detected for FD time (*qP-FD3.1*^*m*^*, −4.1*^*m*^*, −4.2*^*m*^) co-localized with MD QTLs (*qP-MD3.1*^*m*^*, −4.1*^*m*^*, −4.2*^*m*^) detected. Also, two other MD QTLs detected (*qP-MD2.1*^*m*^, *−5.1*^*m*^) also co-localized with BT QTLs (*qP-BT2.1*^*m*^ and *qP-BT5.1*), previously detected in the same plant material^6^. These results confirm the correlation discussed above and indicate that in our sweet cherry, plant material MD mainly depends on the genetic control of BT and FD time. FD time QTLs on LGs 3 and 4 (*qP-FD3.1*^*m*^ and *qP-FD4.2*^*m*^) and BT LG2 QTL (*qP-BT2.1*^*m*^)^6^ are the main determinants of MD in this plant material.

Previously, MD QTLs have also been mapped on LGs 1, 4, and 5 of sweet cherry^7,9^ but not on LGs 2 and 3 as herein. In other *Prunus* species, MD QTLs have been previously reported on LGs 1–7 of peach^10,11,13,14,25,32^ and apricot^5^, and on LG4 of plum^12^. In these works, as detected herein, the main QTL controlling MD and FD time was mapped to a synthetic region on LG4. A *NAC* transcription factor has been reported as the strongest candidate gene for this trait at this QTL in peach^15^. *NAC* transcription factors have been reported to contribute to different plant development and stress- resistance functions^33^. In this work, two *NAC* transcription factors work together (*Pav_sc0000029.1_g070.1.mk* and *Pav_sc0000029.1_g090.1.mk*), within the major FD time, and MD LG4 QTLs were identified in the sweet cherry genome sequence^28^. These genes may be candidates for MD regulation in sweet cherry by regulating the FD time. The same genomic region on LG4 has been associated with FF genetic control earlier^24^ and in this work. As described in other fruit species^16,34,35^, *NAC* transcription factors may also be associated with FF variability in sweet cherry. *NAC* transcription factors in peach and apple^11,15,16^ have also been associated with softer fruits of early ripening. *NAC* transcription factors identified in this work at LG4 have also been previously co-localized with a MD QTL in sweet cherry^9^. Further analyses are ongoing to characterize these genes, and to identify polymorphisms putatively associated with early- or late-ripening phenotypes.

### FS and firmness

The heritability estimates of FS (0.93) and weight (0.92) were higher than those previously reported in other sweet cherry studies (0.63–0.88)^20,22,23,30^. However, the collection of significantly smaller fruits in 2017 than in 2018 revealed that environmental factors affecting FS varied between years. In addition, both years, skewed distribution to large fruits was observed when analyzing all the populations together. However, FS distribution was skewed toward small fruits in most populations (Fig. 1). This result is consistent with previous observations in sweet cherry that suggest semidominance of small FS^20,22,23^. The very low FS of C×C compared with the other populations biased the distribution to large FS when analyzing all the populations together. FF *H*^2^ (0.84) was in the same range as earlier reported (0.73–0.97)^22–24^, and yearly differences were not observed, indicating that this trait is more stable than size. A normal distribution, as described previously for FF in sweet cherry, was observed in this study^22^. The negative correlation observed between fruit dimension (size and weight) and firmness is highly relevant, as these traits are considered the main drivers of cherry acceptability^36^. The same negative correlation has been observed before^22^; however, in other genetic backgrounds, no correlation or positive correlation between these traits has also been observed^23,29,30^. The results indicate different alleles in different plant material^23^.

All major FS QTLs found previously using single populations were detected in this work. FS QTLs previously identified in sweet cherry mapped to LGs 2 and 6^20,21^, LG5^22^, and LG1^23^. Herein, we identified four QTLs for FS and weight on LGs 1, 2, and 5 (*qP-FW1.1*^*m*^, *qP-FW2.1*^*m*^, *qP-FS2.1*^*m*^, and *qP-FW5.1*^*m*^) co-localizing with previously reported FS QTLs^20–23^. The most significant QTLs for FS and weight (*qP-FW2.1*^*m*^ and *qP-FS2.1*^*m*^) detected herein overlap with previously reported QTLs on LG2^20,21^, thus validating this LG2 region as the main determinant of FS in sweet cherry. The physical position spanned by FW QTL *qP-FW1.1*^*m*^ (11.08–30.61 Mbp) also corresponds to the same region where a cluster of FS and firmness QTLs were mapped from ‘Ambrunés’^23^. Similarly, the LG5 region (8.34–13.18 Mbp) of FS QTLs (*qP-FW5.1*^*m*^ and *qP-FS5.1*) overlaps with FW QTLs detected previously^22,23^. The validation in this work of the main FS QTLs detected previously in sweet cherry highlights the potential of the multifamily QTL approach to investigate the genetics of quantitative traits.

The major FF QTL identified on LG4 (*qP-FF4.1*^*m*^) maps to the same position as the major QTL recently reported by Cai et al.^24^ (*qP-FF4.1*) for the same trait. The haplotype analysis of *qP-FF4.1* (ref. ^24^) revealed that most bred cultivars carried firm alleles for this QTL, whereas only mazzards were homozygous for soft alleles^24^. These results revealed selection of firm alleles at this QTL during cherry domestication^24^. These results also explain why this major QTL had not been detected in other works with other plant materials^22,23^. However, the inclusion in this work of ‘Cristobalina’, a landrace with a firm/soft genotype at this QTL (*qP-FF4.1*^*m*^), has also allowed the detection of this major QTL. This QTL was segregating in the populations analyzed, and was therefore detected in this work. Another firmness QTL detected here with minor effects on LG6 (*qP-FF6.1*^*m*^) has also been previously detected in sweet cherry^22–24^, also exhibiting a lower PVE, revealing the presence of other genes having minor effects on FF.

### SSCs and TA

In this work, as earlier reported in cherry^30^ and peach^14,37,38^, moderate *H*^2^ was observed for SSC (0.62) and TA (0.54). Not a common pattern in SSC or TA values was observed in the parental cultivars, confirming the lower heritability and larger dependence on the environment of these traits^39^. Normal distribution was observed for SSC and TA herein, as previously observed in other peach populations for the same traits^10,25^, revealing the quantitative nature of these traits.

QTLs for SSC and TA are first reported for sweet cherry in this study. Previous QTL analyses in apricot, peach, and plum of these traits reported a large number of QTLs^4,10,12,25–27^. In peach, TA QTLs have been reported on LGs 2, 3, 4, 5, and 6^4,10,25,26^. A major locus (*D*), mapped on LG5, has been reported as the major determinant of acid and subacid fruit taste in peach^25,40^. This LG5 QTL was not detected in this work for sweet cherry. On the other hand, major TA QTL, detected in this work on LG6 (*qP-TA6.1*^*m*^), was also reported in a homologous region in peach (*qTA6.2*)^14^. For SSC, the main QTL found in this work on LG4 (*qP-SSC4.1*^*m*^) also overlaps with a peach QTL for this trait^4,14,25,26^, suggesting that there may be a common path regulating this trait in both species.

### Phenology and fruit quality determined by LG4

A correlation between phenology and fruit-quality traits was observed in this work. The positive correlation observed for MD and FD time, with FF and SSC, confirms previous results in other sweet cherry genetic backgrounds, in which higher firmness and SSC are observed in genotypes with later maturity^29,30^. Traditional varieties, such as ‘Ambrunés’, with late-ripening date and large FD time period, have higher SSC and FF than varieties of early ripening^41^. These results may confirm previous studies that indicate that SSC is related to photoassimilation, and cultivars with long FD time are expected to accumulate larger SSC than those with shorter periods^42^.

In this study, stable and major QTLs for MD, FD time, FF, and SSC were identified overlapping in a narrow region of LG4 (50–59 cM; 10.41–12.57 Mbp). In addition, a significant correlation between some of these traits was observed in this work. A cluster of QTLs was also reported on the homologous region of peach^10,25,26^, apricot^27^, and plum^12^ genetic maps for related traits. In apple, Kenis et al.^43^ mapped a large number of QTLs, for the same traits, on LG10, in a syntenic region to LG4 of *Prunus* species^44^. Therefore, a conserved region in some *Rosaceae* species determines the main phenology and fruit-quality traits. The correlation and common physical location of these traits may be due to multiple linked genes, or to a major gene for MD with a pleiotropic effect on the other fruit-quality traits^25^. This major MD determinant may be a FD time determinant, and correlated SSC and firmness variations may be a consequence of differences in FD time in different genotypes. During ripening, fruits accumulate sugars, acids, and other volatile compounds, and cultivars with shorter FD time period may not complete their physiological maturation as much as cultivars with long FD time.

The investigation of this LG4 region (50–54 cM) is of interest for breeding, as selection of certain haplotypes of this genomic region will allow the selection of various phenology and fruit-quality traits at the same time. Haplotypes identified in this region in this work are the same as previously reported for a major FF QTL on the analogous region of LG4 in sweet cherry^24^. Haplotypes *H4-a*, *-b*, *-c*, and *-d* identified in this work for FD time, MD, and FF correspond to FF haplotypes H1, H4, H9, and H6, respectively, identified by Cai et al.^24^. In such work^24^, only haplotype H9 (corresponding to *H4-c* in this work) was identified in ‘Cristobalina’, and as herein, it was associated with low firmness^24^. In the plant material analyzed in this work, ‘Cristobalina’ and ‘Burlat’, both originally from Southern Europe^45^, have an allele associated with short FD time and early MD (*H4-c*). Both are cultivars of early maturity, and the presence of this haplotype may explain this phenotype. However, the same haplotype *H4-c* is also associated with softer fruits in their progenies. As we have seen that early maturity is associated with short FD time, it may be that soft fruits are also associated with short FD time period. In any case, breeding for early fruit will result in soft fruits from this plant material, revealing a complex scenario for the breeding of firm and early fruits from these materials. However, as in this plant material, BT and FD time that are mainly determined by different loci, selecting for specific alleles for the BT QTLs on LGs 1 and 2^6^ and/or FD time QTL on LG4 (this work), may allow obtaining early firm fruits, by selecting early BT and large FD time. The other haplotypes identified in this region of LG4 (*H4-a*, *-b*, *-d*), which are associated with longer FD time, can also be selected for late maturity, firmer fruits, and higher SSC content.

## Conclusions

In this work, the use of multiple sweet cherry populations derived from parental cultivars of different genetic backgrounds showing large phenotypic variability has provided valuable information about the genetic control of phenology and fruit-quality traits. This information will be useful for breeding and for broadening the understanding of the genetics of these traits. Correlation and genetic analyses showed that BT and ripening period are independent, and that MD is dependent on both of them, but on ripening time to a much larger extent. This knowledge will allow the design of specific breeding strategies for specific adaptation and fruit-quality objectives. Most previously reported QTLs for the analyzed traits were validated in this work, and new major QTLs were reported. Of these, the most relevant is a region on LG4 with the presence of highly significant and stable QTLs for FD period, MD, FF, and SSCs. This QTL region overlaps with a region reported in other Rosaceae species where a *NAC* transcription factor has been associated with MD and fruit softening, which represents a target region for marker-assisted breeding. *NAC* transcription factor genes were also identified in the sweet cherry genome in this work, in the same region, and may be candidate genes for the regulation of these traits in sweet cherry. In addition for this LG4 QTL region, specific sweet cherry haplotypes of breeding interest were identified. These will allow selecting for early or late maturity, high or low firmness, and soluble solids in this and related plant material.

## Materials and methods

### Plant materials

In this work, 411 sweet cherry genotypes from six full-sib populations (*N* = 406), the parental cultivars (*N* = 6), and some ancestors (*N* = 5) were used^6^. This plant material includes four cross-pollination populations (F_1_), ‘Vic’ × ‘Cristobalina’ (V×C, *N* = 158), ‘Ambrunés’ × ‘Cristobalina’ (A×C, *N* = 40), ‘Brooks’ × ‘Cristobalina’ (B×C, *N* = 29), and ‘Lambert’ × ‘Cristobalina’ (L×C, *N* = 14), and two self-pollination populations (F_2_). One F_2_ comes from ‘Cristobalina’ self-pollination (C×C, *N* = 97), and the other from the self-pollination of selection ‘BC8’ (B×C F2, *N* = 68). All the plant materials are found at CITA de Aragón orchards (Zaragoza, Spain).

### Trait phenotyping

Phenotype data for seven agronomical and fruit-quality traits were evaluated during 2 years (2017 and 2018) in all the plant material. The traits evaluated were MD, FD time, FS, FW, FF, fruit TA, and fruit SSC. BT data of the same plant material, the same 2 years (data previously reported in Calle et al.^6^), were used for the estimation of FD and for correlation tests.

MD was recorded in calendar days from January 1st as the date when 50% of fruits reached the optimum ripening stage based on visual inspection of fruit color, taste, and firmness. FD was estimated as the days between BT and MD. Fruit-quality traits (size, weight, and firmness) were measured from the same 15 fruits harvested from each tree. FS was measured perpendicular to suture axis using a caliper. FF was assessed on two opposite mediolateral axes using DuroColor^®^ texture analyzer (Setop Giraud Technologie, Cavaillon, France). TA was determined by titrating 5 g of fruit juice from the 15 sampled fruits, with NaOH 0.1 N to pH 8.1 (ref. ^46^) using an automatic titrator (Metrohm, Herisau, Swiss). Using the same fruit juice, soluble solid contents (SSC) were determined using a refractometer (Atago, Tokyo, Japan) and the data were presented in °Brix. For these data, minimum and maximum values and histograms for each trait and population were checked to identify outliers that could be derived from errors during phenotyping.

### Statistical analysis of phenotypic data

Statistical analyses of data were performed to estimate the mean, minimum, maximum, and standard deviation in each population per year and trait. Mean comparison (ANOVA and Tukey test, *p* < 0.05) of parental values was carried out for FS, weight, and firmness (traits measured in 15 fruits per tree and year). Correlation between traits each year was analyzed using the *Spearman* correlation coefficient. Normal distribution was tested per trait and year using the Shapiro–Wilk test (*p* < 0.05). Broad-sense heritability (*H*^2^) for each trait was calculated using the equation: *H*^2^ = $\frac{{\sigma _g^2}}{{\sigma _g^2 + \frac{{\sigma _e^2}}{n}}}$, where $\sigma _g^2$ is the variance of genotype effect, $\sigma _e^2$ is the variance of the residual term, and *n* is the number of years. All statistical analyses and figures were performed using ‘*psych’*, ‘*ggplot’*, and ‘*corrplot’* packages of R v3.4.1 (ref. ^47^).

### QTL analysis and haplotype construction

Plant material used in this study has been previously genotyped with RosBREED cherry 6 K SNP array v1 (ref. ^48^), and the genotypic data set was previously prepared^6^. For QTL analyses, Bayesian multiple QTL model implemented by FlexQTL^™^^49,50^ software was used. FlexQTL^™^ model was settled to consider up to a maximum of 10 QTLs per simulation and additive QTL effects with a normal prior distribution and random (co)variance matrix with diagonal structure. Preliminary runs with dominant effect models were performance, and no differences in the results from additive models were observed. Whole-genome QTL analysis was carried out four times for each trait varying prior number of QTLs (1 and 3) and seed numbers to create independence between iterations. For each simulation, Markov chain Monte Carlo (MCMC) simulations with minimum of 500,000 iterations were performed until at least 100 effective chain samples for the overall mean, the residual variance, the number of QTLs, and the variance of this number^50^. A detailed information of genetic model settings and statistical parameters considered for QTL analysis using FlexQTL^™^ is shown in Supplementary Table 5. Only data from one simulation (prior number of QTLs set to 1) for each year and trait were reported in this study. The two times the natural log of *Bayes factors* (*BF*) was used to determine the number and position of QTLs. Only QTLs with strong and decisive evidences (2*lnBF* > *5* and *10*, respectively) were reported. The graphical representations of LGs and QTLs were obtained using MapChart software^51^.

Parental and ancestor haplotypes were constructed for major stable FD, MD, and FF QTL on LG4 (50–54 cM). Haplotypes were obtained from SNP phase estimated by FlexQTL^™^. Mean phenotypic values for each segregating class of each population for those individuals without recombination events in this genomic region (LG4 50–54 cM) were estimated for these three traits (FD, MD, and FF) and for SSC. Mean comparison among phenotypic values of each segregating class within each population was estimated by ANOVA, Kruskal–Wallis, and two-tailed Student’s test (*p* < 0.01). Statistical analyses were carried out using IBM SPSS v21 (Chicago, IL, USA).

### Candidate gene search in sweet cherry genome

To investigate candidate gene colocalization in major QTLs, *NAC* transcription factors were searched in FD and MD QTL on LG4 (51–54 cM). *NAC* transcription factors have been reported as candidate genes for fruit maturity^15,34,35^. Genes annotated as *NAC* transcription factors in ‘Satonishiki’ sweet cherry genome (PAV_r1.0, ref. ^28^) physical region corresponding to 51–53 cM on LG4 (chr4: 13,304,990–14,860,789 bps) were searched for.

## Supplementary information

Supplementary information

## Data Availability

The datasets generated for this study can be found in the Genome database for Rosaceae (https://www.rosaceae.org/publication_datasets). Accession no. tfGDR1041.
