# Supplementary material for: Multiple-population QTL mapping of maturity and fruit-quality traits reveals LG4 region as a breeding target in sweet cherry (Prunus avium L.)
Source: Hortic Res. 2020 Aug 1;7:127. doi: 10.1038/s41438-020-00349-2 (PMC7395078; doi:10.1038/s41438-020-00349-2)
Supplement: Supplementary file 1 — Supplementary information [file 41438_2020_349_MOESM1_ESM.docx]

**Supplementary Table 1** Minimum (Min), maximum (Max), mean and standard deviation (SD) values of fruit development time (FD), maturity date (MD), fruit weight (FW), fruit size (FS), fruit firmness (FF), soluble solid content (SSC), and titratable acidity (TA) of parental cultivars (a) and populations (b), in years 2017 and 2018. The number of individuals phenotyped each year and Spearman correlation coefficient between both years is also included for the populations (b).

**a)**

|  |  | **FD (Days)** | | **MD (Days)** | | **FW (g)** | | **FS (mm)** | | **FF (%)** | | **SSC (ºBrix)** | | **TA (%)** | |
| --- | --- | --- | --- | --- | --- | --- | --- | --- | --- | --- | --- | --- | --- | --- | --- |
|  |  | **2017** | **2018** | **2017** | **2018** | **2017** | **2018** | **2017** | **2018** | **2017** | **2018** | **2017** | **2018** | **2017** | **2018** |
| **‘Ambrunés’** |  | 77 | 77 | 153 | 171 | 7.63 b | 8.99 b | 24.69 b | 26.12 b | 54.9 b | 48.6 b | 18.7 | 18.8 | 0.81 | 0.57 |
| **‘Brooks’** |  | 64 | 57 | 142 | 155 | 7.97 b | 9.07 b | 26.07 b | 27.59 b | 52.1 b | 55.6 c | 19.8 | 19.1 | 0.75 | 0.78 |
| **‘Cristobalina’** | | 58 | 60 | 122 | 138 | 4.01 a | 4.15 a | 19.5 a | 19.69 a | 63.4 a | 52.9 bc | 17.0 | 21.0 | 0.60 | 0.71 |
| **‘Lambert’** |  | 78 | 72 | 163 | 173 | 9.24 c | 8.65 b | 25.41 b | 25.46 b | 47.3 c | 33.1 a | - | 18.8 | - | 0.76 |
| **‘Vic’** |  | 71 | 68 | 153 | 169 | 9.42 c | 9.23 b | 26.28 b | 26.31 b | 46.1 c | 50.3 bc | 20.6 | 21.3 | 0.88 | 0.71 |

**Supplementary Table 1** Continued.

**b)**

|  |  | **Fruit Dev. Time (Days)** | | **Maturity Date (Days)** | | **Fruit Weight**  **(g)** | | **Fruit Size**  **(mm)** | | **Fruit Firmness (%)** | | **SSC**  **(ºBrix)** | | **Titratable Acidity (%)** | | | **Nr. individuals**  **phenotyped**  **each year** | | **Nr. individuals**  **phenotyped**  **both years** |
| --- | --- | --- | --- | --- | --- | --- | --- | --- | --- | --- | --- | --- | --- | --- | --- | --- | --- | --- | --- |
|  |  | **2017** | **2018** | **2017** | **2018** | **2017** | **2018** | **2017** | **2018** | **2017** | **2018** | **2017** | **2018** | **2017** | **2018** | **2017** | | **2018** |  |
| **A×C** | **Min** | 58 | 53 | 135 | 148 | 3.3 | 3.9 | 18.1 | 20.0 | 31.2 | 38.2 | 16.7 | 15.2 | 0.61 | 0.51 | 31 | | 39 | 31 |
|  | **Max** | 78 | 83 | 153 | 173 | 7.2 | 8.1 | 24.9 | 26.3 | 71.2 | 75.6 | 29.7 | 27.5 | 1.12 | 0.97 |  | |  |  |
|  | **Mean** | 69.8 | 68.7 | 146 | 161.2 | 4.8 | 5.6 | 20.8 | 22.3 | 54.3 | 55.9 | 21.9 | 19.1 | 0.86 | 0.73 |  | |  |  |
|  | **SD** | 5.5 | 8.0 | 5.4 | 8.2 | 0.9 | 1.1 | 1.4 | 1.6 | 11.0 | 9.9 | 3.7 | 2.4 | 0.12 | 0.11 |  | |  |  |
| **B×C** | **Min** | 49 | 55 | 124 | 144 | 4.03 | 5.83 | 20.0 | 22.7 | 27.6 | 31.1 | 14.0 | 16.4 | 0.55 | 0.51 | 17 | | 13 | 12 |
|  | **Max** | 75 | 75 | 150 | 166 | 7.24 | 8.62 | 24.8 | 26.5 | 59.8 | 67.4 | 26.1 | 21.2 | 0.97 | 0.89 |  | |  |  |
|  | **Mean** | 64.5 | 66 | 138.6 | 157.8 | 6.0 | 7.0 | 22.9 | 24.4 | 50.1 | 52.5 | 20.4 | 18.5 | 0.72 | 0.67 |  | |  |  |
|  | **SD** | 8.7 | 5.9 | 9.5 | 6.4 | 0.9 | 0.9 | 1.3 | 1.2 | 7.3 | 12.4 | 3.1 | 1.5 | 0.12 | 0.12 |  | |  |  |
| **B×C F2** | **Min** | 53 | 48 | 125 | 141 | 3.44 | 3.19 | 19.0 | 19.2 | 26.2 | 15.2 | 17.1 | 14.9 | 0.71 | 0.65 | 19 | | 27 | 19 |
|  | **Max** | 79 | 75 | 151 | 168 | 6.33 | 7.21 | 24.3 | 25.3 | 59.0 | 77 | 25.1 | 22.4 | 1.01 | 1.13 |  | |  |  |
|  | **Mean** | 63.4 | 59.2 | 139.3 | 153.7 | 4.8 | 5.4 | 21.2 | 22.4 | 46.5 | 48.2 | 19.9 | 18.9 | 0.9 | 0.89 |  | |  |  |
|  | **SD** | 7.9 | 8.3 | 7.7 | 7.2 | 0.7 | 0.7 | 1.2 | 1.2 | 8.7 | 13.7 | 2.0 | 1.6 | 0.09 | 0.13 |  | |  |  |
| **C×C** | **Min** | 53 | 53 | 120 | 138 | 2.27 | 2.03 | 16.4 | 15.2 | 48.0 | 49 | 16.6 | 18.7 | 0.43 | 0.67 | 19 | | 26 | 16 |
|  | **Max** | 77 | 76 | 144 | 158 | 4.45 | 4.61 | 20.5 | 20.6 | 86.7 | 87.4 | 24.1 | 24.4 | 0.95 | 1.17 |  | |  |  |
|  | **Mean** | 62.8 | 63.8 | 129.5 | 145.6 | 3.0 | 3.1 | 18.0 | 18.1 | 68.2 | 67.7 | 20.3 | 21.2 | 0.69 | 0.89 |  | |  |  |
|  | **SD** | 7.1 | 6.3 | 7.2 | 4.9 | 0.6 | 0.6 | 1.3 | 1.3 | 10.0 | 8.8 | 2.6 | 1.7 | 0.15 | 0.12 |  | |  |  |
| **L×C** | **Min** | 57 | 55 | 132 | 151 | 3.52 | 3.77 | 18.2 | 18.5 | 29.9 | 40.1 | 17.3 | 15.9 | 0.70 | 0.80 | 11 | | 10 | 8 |
|  | **Max** | 79 | 75 | 158 | 169 | 7.12 | 8.57 | 24.5 | 26.2 | 78.4 | 68.3 | 25.1 | 25.7 | 1.28 | 1.20 |  | |  |  |
|  | **Mean** | 65.9 | 67.2 | 145.3 | 162.1 | 5.8 | 6.5 | 22.0 | 23.3 | 50.6 | 54.7 | 20.2 | 19.6 | 0.92 | 0.93 |  | |  |  |
|  | **SD** | 7.4 | 6.6 | 8.8 | 6.6 | 1.2 | 1.6 | 2.0 | 2.6 | 16.6 | 9.9 | 2.8 | 3.5 | 0.17 | 0.13 |  | |  |  |
| **V×C** | **Min** | 50 | 48 | 127 | 142 | 4.08 | 5.07 | 19.0 | 20.9 | 33.0 | 28.7 | 17.3 | 14.6 | 0.62 | 0.49 | 99 | | 141 | 95 |
|  | **Max** | 84 | 77 | 163 | 173 | 7.54 | 9.38 | 24.9 | 26.6 | 74.7 | 81.2 | 28.5 | 26.3 | 1.07 | 1.17 |  | |  |  |
|  | **Mean** | 66 | 63.8 | 144.5 | 159.3 | 5.8 | 7.2 | 22.5 | 24.3 | 54.0 | 53.3 | 23.1 | 20.2 | 0.89 | 0.79 |  | |  |  |
|  | **SD** | 6.4 | 7.6 | 6.7 | 8.0 | 0.7 | 0.8 | 1.0 | 1.1 | 9.8 | 10.4 | 2.8 | 2.2 | 0.11 | 0.13 |  | |  |  |
| **Spearman Correlation Coeff (2017-2018)** | | 0.84 |  | 0.91 |  | 0.83 |  | 0.82 |  | 0.73 |  | 0.54 |  | 0.46 |  |  | |  |  |

**Supplementary Table 2** QTLs identified both years.

|  | **Year** | **Identified both years?** | **QTL name** | **QTL Evidences** | **LG** | **Interval (cM)** | **QTL peak (cM)** | **Physical poisiton* (Mbp)** | **Max 2lnBF** | **Average 2lnBF** | **Mean Additive effect** | **PVE (%)** | **Total Phenotype Variance Explained by QTLs** |
| --- | --- | --- | --- | --- | --- | --- | --- | --- | --- | --- | --- | --- | --- |
| **Fruit Development Time (FD)** | 2017 | Yes | *qP-FD3.1^m^* | Strong | 3 | 17-62 | 45 | 4.16-19.65 | 5.08 | 3.44 | 2.77 | 2.66 | 73.83 |
|  | 2017 | Yes | *qP-FD4.1^m^* | Strong | 4 | 9-32 | 21 | 2.15-6.82 | 5.31 | 3.93 | 5.07 | 5.85 |  |
|  | 2017 | Yes | *qP-FD4.2^m^* | Decisive | 4 | 51-53 | 53 | 10.88-11.66 | 14.04 | 11.69 | 10.77 | 65.32 |  |
|  | 2018 | No | *qP-FD1.1* | Strong | 1 | 44-65 | 59 | 11.78-23.59 | 8.59 | 5.72 | 2.51 | 3.67 | 93.65 |
|  | 2018 | Yes | *qP-FD3.1^m^* | Decisive | 3 | 25-41 | 35 | 6.02-10.88 | 10.93 | 6.24 | 3.12 | 6.91 |  |
|  | 2018 | Yes | *qP-FD4.1^m^* | Decisive | 4 | 8-30 | 17 | 1.98-6.58 | 10.16 | 5.93 | 5.74 | 18.16 |  |
|  | 2018 | Yes | *qP-FD4.2^m^* | Decisive | 4 | 51-53 | 53 | 10.88-11.66 | 14.02 | 11.77 | 11.66 | 64.53 |  |
|  | 2018 | No | *qP-FD5.1* | Strong | 5 | 59-67 | 61 | 13.70-16.87 | 6.08 | 5.22 | 1.83 | 0.38 |  |
| **Maturity Day (MD)** | 2017 | Yes | *qP-MD1.1^m^* | Strong | 1 | 56-77 | 67 | 18.54-28.94 | 6.31 | 3.83 | 3.35 | 5.39 | 89.65 |
|  | 2017 | Yes | *qP-MD2.1^m^* | Decisive | 2 | 68-76 | 75 | 25.24-29.94 | 11.67 | 6.08 | 3.88 | 11.71 |  |
|  | 2017 | Yes | *qP-MD3.1^m^* | Strong | 3 | 13-52 | 43 | 3.70-15.84 | 5.17 | 3.49 | 5.37 | 19.51 |  |
|  | 2017 | Yes | *qP-MD4.1^m^* | Strong | 4 | 5-33 | 15 | 1.98-6.82 | 4.06 | 2.71 | 3.87 | 4.18 |  |
|  | 2017 | Yes | *qP-MD4.2^m^* | Decisive | 4 | 51-53 | 53 | 10.88-11.66 | 12.07 | 9.53 | 11.14 | 46.78 |  |
|  | 2017 | Yes | *qP-MD5.1^m^* | Strong | 5 | 57-71 | 69 | 13.62-18.41 | 6.16 | 4.73 | 2.18 | 2.1 |  |
|  | 2018 | Yes | *qP-MD1.1^m^* | Strong | 1 | 50-63 | 57 | 14.33-23.45 | 8.92 | 7.59 | 4.06 | 8.62 | 92.06 |
|  | 2018 | Yes | *qP-MD2.1^m^* | Decisive | 2 | 73-76 | 75 | 26.96-29.94 | 15.15 | 11.67 | 4.93 | 10.4 |  |
|  | 2018 | Yes | *qP-MD3.1^m^* | Strong | 3 | 31-42 | 33 | 7.50-10.88 | 9.72 | 8.1 | 3.8 | 6.68 |  |
|  | 2018 | Yes | *qP-MD4.1^m^* | Strong | 4 | 8-15 | 11 | 1.93-4.19 | 9.29 | 7.72 | 6.13 | 11.03 |  |
|  | 2018 | Yes | *qP-MD4.2^m^* | Decisive | 4 | 51-53 | 53 | 10.88-11.66 | 14.89 | 11.76 | 11.6 | 52.53 |  |
|  | 2018 | Yes | *qP-MD5.1^m^* | Decisive | 5 | 64-71 | 67 | 15.81-18.41 | 11.18 | 8.15 | 2.32 | 2.81 |  |
| **Weight (FW)** | 2017 | Yes | *qP-FW1.1^m^* | Strong | 1 | 52-74 | 63 | 14.89-27.67 | 6.27 | 5.16 | 0.81 | 6.07 | 83.73 |
|  | 2017 | Yes | *qP-FW2.1^m^* | Strong | 2 | 31-74 | 39 | 17.86-28.60 | 7.53 | 4.87 | 0.98 | 23.95 |  |
|  | 2017 | No | *qP-FW4.1* | Strong | 4 | 43-61 | 51 | 9.15-13.61 | 9.06 | 5.31 | 0.4 | 3.39 |  |
|  | 2017 | Yes | *qP-FW5.1^m^* | Strong | 5 | 31-49 | 35 | 8.42-12.39 | 9.34 | 6.51 | 1.45 | 45.42 |  |
|  | 2017 | No | *qP-FW6.1* | Strong | 6 | 13-33 | 23 | 4.17-7.26 | 9.18 | 6.01 | 0.39 | 4.89 |  |
| **Supplementary Table 2** Continued. | | | | | | | | | | | | | |
|  | 2018 | Yes | *qP-FW1.1^m^* | Strong | 1 | 42-84 | 71 | 11.08-30.61 | 5.52 | 4.49 | 1.14 | 15.73 | 78.84 |
|  | 2018 | Yes | *qP-FW2.1^m^* | Strong | 2 | 45-73 | 67 | 21.72-27.46 | 8.96 | 5.93 | 1.73 | 53.9 |  |
|  | 2018 | No | *qP-FW3.1* | Strong | 3 | 19-52 | 29 | 4.45-15.84 | 7.33 | 4.84 | 0.43 | 2.27 |  |
|  | 2018 | Yes | *qP-FW5.1^m^* | Strong | 5 | 39-54 | 49 | 11.20-13.18 | 5.46 | 4.37 | 1.28 | 6.94 |  |
| **Size (FS)** | 2017 | Yes | *qP-FS2.1^m^* | Strong | 2 | 57-76 | 65 | 23.74-29.94 | 8.99 | 6.75 | 1.43 | 23.59 | 75.06 |
|  | 2017 | No | *qP-FS5.1* | Strong | 5 | 30-50 | 35 | 8.34-12.39 | 9.2 | 4.93 | 2.18 | 44.56 |  |
|  | 2017 | No | *qP-FS6.1* | Strong | 6 | 15-31 | 25 | 4.63-7.06 | 9.94 | 6.88 | 0.73 | 6.92 |  |
|  | 2018 | No | *qP-FS1.1* | Strong | 1 | 45-71 | 55 | 12.30-26.89 | 7.31 | 5.31 | 1.76 | 22.21 | 48.07 |
|  | 2018 | Yes | *qP-FS2.1^m^* | Decisive | 2 | 63-73 | 71 | 24.83-27.46 | 10.96 | 7.21 | 1.06 | 21.49 |  |
|  | 2018 | No | *qP-FS4.1* | Strong | 4 | 10-46 | 21 | 2.55-10.08 | 6.83 | 5.11 | 0.78 | 4.37 |  |
| **Firmness (FF)** | 2017 | Yes | *qP-FF4.1^m^* | Decisive | 4 | 50-54 | 51 | 10.41-12.57 | 12.15 | 11.75 | 14.4 | 47.95 | 63.68 |
|  | 2017 | Yes | *qP-FF6.1^m^* | Strong | 6 | 78-109 | 99 | 23.80-30.45 | 6.86 | 4.83 | 3.66 | 2.54 |  |
|  | 2018 | Yes | *qP-FF4.1^m^* | Decisive | 4 | 51-53 | 51 | 10.88-11.66 | 11.03 | 9.51 | 15 | 64.05 | 86.02 |
|  | 2018 | Yes | *qP-FF6.1^m^* | Strong | 6 | 74-109 | 87 | 22.65-30.45 | 3.36 | 2.27 | 2.86 | 1.31 |  |
| **Soluble solid content (SSC)** | 2017 | Yes | *qP-SSC3.1^m^* | Strong | 3 | 13-40 | 27 | 3.94-10.77 | 9.32 | 4.76 | 1.5 | 10.42 | 44.58 |
|  | 2017 | Yes | *qP-SSC4.1^m^* | Decisive | 4 | 50-55 | 53 | 10.41-12.72 | 14.09 | 11.7 | 3.04 | 34.16 |  |
|  | 2018 | No | *qP-SSC1.1* | Strong | 1 | 37-74 | 63 | 9.77-27.67 | 9.01 | 4.82 | 1.26 | 15.26 | 44.78 |
|  | 2018 | Yes | *qP-SSC3.1^m^* | Strong | 3 | 18-69 | 59 | 4.50-21.85 | 8.29 | 4.19 | 0.89 | 7.41 |  |
|  | 2018 | Yes | *qP-SSC4.1^m^* | Strong | 4 | 45-59 | 53 | 10.08-13.10 | 9.6 | 6.82 | 1.69 | 22.11 |  |
| **Titratable acidity (TA)** | 2017 | No | *qP-TA4.1* | Strong | 4 | 34-64 | 51 | 7.06-14.83 | 7.31 | 4.14 | 0.0561 | 5.39 | 26.97 |
|  | 2017 | Yes | *qP-TA6.1^m^* | Decisive | 6 | 91-98 | 95 | 26.68-27.49 | 11.83 | 9.65 | 0.0941 | 21.57 |  |
|  | 2018 | No | *qP-TA3.1* | Strong | 3 | 72-89 | 87 | 22.74-26.99 | 9.94 | 6.17 | 0.0588 | 5.01 | 20.02 |
|  | 2018 | Yes | *qP-TA6.1^m^* | Decisive | 6 | 91-108 | 97 | 26.77-30.45 | 10.29 | 6.33 | 0.0694 | 15.02 |  |

**Supplementary Table 3** Parental and ancestor haplotypes for FD (*qP-FD4.2^m^*), MD (*qP-MD4.2^m^*) and FF (*qP-FF4.1^m^*) QTLs in LG4 50-54 cM.

| **SNP** | **Genetic position (cM)** | **Physical position (bp)** | **Haplotype** | | | | | | | | | | | | | | | | | | | | | | |
| --- | --- | --- | --- | --- | --- | --- | --- | --- | --- | --- | --- | --- | --- | --- | --- | --- | --- | --- | --- | --- | --- | --- | --- | --- | --- |
|  |  |  | **'Ambrunes'** | | **'BC8'** | | **'Bing'** | | **'Brooks'** | | **'Burlat'** | | **'Cristobalina'** | | **'Lambert'** | | **'Napoleon'** | | **'Rainier'** | | **'Van'** | | **'Vic'** | |  |
|  |  |  | ***H4-a*** | ***H4-a*** | ***H4-d*** | ***H4-c*** | ***H4-b*** | ***H4-b*** | ***H4-b*** | ***H4-d*** | ***H4-c*** | ***H4-d*** | ***H4-c*** | ***H4-a*** | ***H4-b*** | ***H4-b*** | ***H4-a*** | ***H4-b*** | ***H4-b*** | ***H4-b*** | ***H4-b*** | ***H4-a*** | ***H4-b*** | ***H4-a*** |  |
| ss490559054 | 50.96 | 10414884 | B | B | B | B | A | A | A | B | B | B | B | B | A | A | B | A | A | A | A | B | A | B |  |
| ss490552906 | 51.09 | 10880163 | A | A | A | B | A | A | A | A | B | A | B | A | A | A | A | A | A | A | A | A | A | A |  |
| ss490552928 | 53.03 | 11472398 | B | B | A | A | B | B | B | A | A | A | A | B | B | B | B | B | B | B | B | B | B | B |  |
| ss490552931 | 53.12 | 11520743 | A | A | A | B | A | A | A | A | B | A | B | A | A | A | A | A | A | A | A | A | A | A |  |
| ss490548726 | 53.41 | 11661240 | A | A | B | A | A | A | A | B | A | B | A | A | A | A | A | A | A | A | A | A | A | A |  |
| ss490552936 | 53.41 | 11661765 | A | A | B | A | A | A | A | B | A | B | A | A | A | A | A | A | A | A | A | A | A | A |  |

**Supplementary Table 4** Phenotypic mean values and mean comparison of maturity date, fruit development, fruit firmness, and SSC in each segregating class of each population on LG4 50-54 cM interval region. Statistical differences (p<0.05) within classes for families are indicated.

| **Population** | **Detected genotype classes** | **N** | **Maturity Date** | | **Fruit Development** | | **Fruit Firmness** | | **SSC** | |
| --- | --- | --- | --- | --- | --- | --- | --- | --- | --- | --- |
|  |  |  | **2017**  **(Mean ± SD)** | **2018**  **(Mean ± SD)** | **2017**  **(Mean ± SD)** | **2018**  **(Mean ± SD)** | **2017**  **(Mean ± SD)** | **2018**  **(Mean ± SD)** | **2017**  **(Mean ± SD)** | **2018**  **(Mean ± SD)** |
| **A×C** | *a / a* | 18 | 148.9 ± 4.0 a | 167.1 ± 6.2 a | 72.4 ± 4.2 a | 74.7 ± 5.6 a | 62.3 ± 6.3 a | 62.1 ± 9.6 a | 23.1 ± 4.4 a | 20.2 ± 2.8 a |
|  | *a / c* | 20 | 142.5 ± 4.7 b | 155.9 ± 5.9 b | 66.4 ± 5.2 b | 63.3 ± 5.8 b | 45.3 ± 7.8 b | 50.8 ± 6.8 b | 20.8 ± 2.7 b | 18.1 ± 1.6 b |
| **B×C** | *a / b* | 9 | 143.0 ± 5.8 a | 160.3 ± 3.0 a | 69.7 ± 4.5 ab | 69.3 ± 3.9 a | 55.0 ± 3.4 | 61.7 ± 5.7 a | 20.9 ± 2.3 ab | 17.4 ± 0.8 |
|  | *b / c* | 8 | 133.5 ± 10.5 b | 152.0 ± 8.0 b | 60.0 ± 8.4 ab | 61.5 ± 6.6 b | 44.9 ± 9.1 | 44.1 ± 8.7 b | 19.3 ± 3.3 ab | 18.2 ± 1.5 |
|  | *a / d* | 6 | 147.0 ± 1.7 a | 163.3 ± 2.3 b | 71.3 ± 2.3 b | 70.3 ± 3.2 a | 51.5 ± 4.6 | 62.3 ± 4.1 a | 23.7 ± 2.1 b | 20.1 ± 1.6 |
|  | *c / d* | 5 | 128.5 ± 4.9 b | 156.0 ± 2.8 ab | 52.5 ± 4.9 a | 62.0 ± 2.8 b | 48.6 ± 4.9 | 36.3 ± 7.3 b | 17.3 ± 1.3 a | 19.5 ± 1.6 |
| **B×C F2** | *c / c* | 11 | - | 146.5 ± 7.8 a | - | 50.0 ± 2.8 a | - | 33.5 ± 5.8 a | - | 18.4 ± 1.0 |
|  | *c / d* | 32 | 134.4 ± 5.4 a | 150.3 ± 4.3 a | 57.9 ± 6.2 a | 55.1 ± 3.6 a | 45.1 ± 4.2 a | 44.1 ± 5.0 b | 19.4 ± 1.4 a | 18.9 ± 1.5 |
|  | *d / d* | 18 | 145.9 ± 4.1 b | 162.1 ± 4.1 b | 71.9 ± 4.8 b | 70.6 ± 3.1 b | 53.0 ± 7.0 b | 64.8 ± 7.9 c | 21.2 ± 2.5 b | 19.8 ± 1.4 |
| **C×C** | *a / a* | 11 | 138.4 ± 3.4 b | 150.7 ± 4.0 b | 72.6 ± 2.9 b | 70.9 ± 3.9 b | 71.2 ± 7.8 a | 77.7 ± 7.1 a | 21.9 ± 1.3 a | 20.7 ± 2.0 |
|  | *a / c* | 56 | 126.2 ± 5.3 a | 144.4 ± 3.5 a | 59.2 ± 4.3 a | 60.9 ± 3.6 a | 66.7 ± 11.1 b | 63.7 ± 6.6 b | 19.4 ± 2.8 b | 21.4 ± 1.6 |
|  | *c / c* | 17 | - | - | - | - | - | - | - | 22.5 ± 2.7 |
| **L×C** | *a / b* | 7 | 153.0 ± 3.2 a | 165.4 ± 4.4 a | 72.6 ± 4.0 a | 70.9 ± 3.1 a | 65.6 ± 8.7 a | 59.9 ± 6.2 a | 23.2 ± 2.8 a | 20.6 ± 3.7 a |
|  | *b / c* | 6 | 138.8 ± 5.9 b | 154.3 ± 3.1 b | 60.3 ± 3.8 b | 58.7 ± 3.2 b | 38.2 ± 8.9 b | 42.5 ± 2.5 b | 18.6 ± 1.1 b | 17.4 ± 2.1 b |
| **V×C** | *a / a* | 30 | 148.8 ± 3.2 a | 165.4 ± 4.7 a | 70.6 ± 3.0 a | 69.9 ± 3.6 a | 58.6 ± 6.6 a | 60.8 ± 7.0 a | 24.5 ± 1.7 a | 20.9 ± 2.0 a |
|  | *a / c* | 41 | 137.4 ± 4.5 b | 152.1 ± 4.6 b | 58.6 ± 3.8 b | 56.9 ± 4.0 b | 44.8 ± 6.6 b | 46.3 ± 7.7 b | 20.7 ± 2.3 b | 19.2 ± 1.7 b |
|  | *a / b* | 41 | 149.2 ± 3.4 a | 167.3 ± 3.5 a | 70.5 ± 3.0 a | 71.6 ± 3.2 a | 61.0 ± 6.3 a | 62.3 ± 5.7 a | 24.7 ± 2.1 a | 21.9 ± 2.1 a |
|  | *b / c* | 40 | 139.6 ± 6.0 b | 154.3 ± 4.9 b | 61.4 ± 4.8 b | 58.7 ± 4.1 b | 45.7 ± 6.8 b | 45.4 ± 4.8 b | 21.5 ± 2.6 b | 18.9 ± 1.7 b |

**Supplementary Table 5** Parameters and data settings used for QTL analyses with FlexQTL^™^ software.

| **Data settings** | | | |
| --- | --- | --- | --- |
| **Parameter** | **Parameter description** | **Considered value** | **Author’s note** |
| **indiN** | Indicator for nuisance variable | 3 | All individuals with genetic information was included in the outputs |
| **nchrom** | Number of chromosomes | 8 |  |
| **indiC** | Chromosomes included in the QTL analysis | 1 | All chromosomes were included for analysis |
| **nmrkrC** | Number of markers per linkage group | n |  |
| **Genetic model settings** | | | |
| **Parameter** | **Parameter description** | **Considered value** | **Author’s note** |
| **indicQTL** | QTL or IBD analysis | 1 | QTL analysis was performed |
| **maximQTL** | Maximum number of QTLs included in the model | 10 |  |
| **priorQTL** | Prior distribution for the number of QTLs | 1 and 3 |  |
| **addGENE** | Additive genetic effects for individual QTL | 5 | Additive genetic model with normal prior distribution and random (Co) variance matrix diagonals |
| **domGENE** | Dominant genetic effects for individual QTL | 0 | Dominance model was excluded from analysis. Preliminary simulations including dominance model were conducted and no differences were observed with additive genetic model. |
| **Statistical parameters** | | | |
| **Parameter** | **Parameter description** | **Considered value** | **Author’s note** |
| **Length** | Length of Markov chains | 500000 |  |
| **Thinning** | Thinning of Markov chain with respect to writing samples to file | 500 |  |
| **ESS** | Effective sample size to obtain a sufficient number of samples for statistical inference | 101 |  |
| **DistanceQTLs** | Minimal distance between QTLs | 0.1 |  |
| **MSegDelta** | Enable or disable extreme segregation distortion for marker loci | 1 | Extreme distortion was accepted |
| **DeleteDR** | Deleting all observed singletons | 0 | Markers causing singletons were maintained |
| **Seed** | Value to start the simulation process in the initial model of the MCMC | Random | Two different simulations were conducted for each analysis with different seed values editing the last digit of seed (0-4 in the first simulation and 5-9 in the second one) to examine convergence of results |


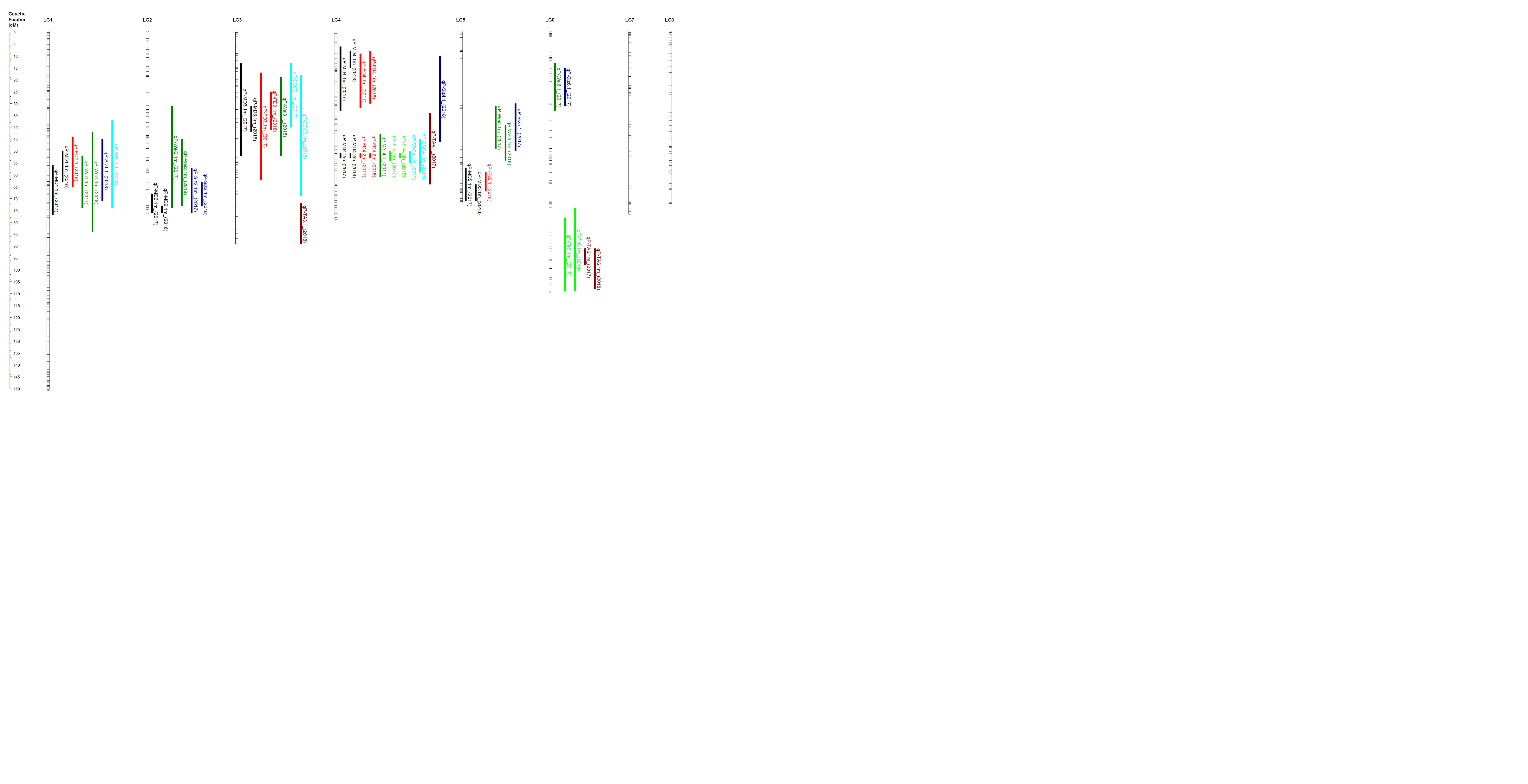


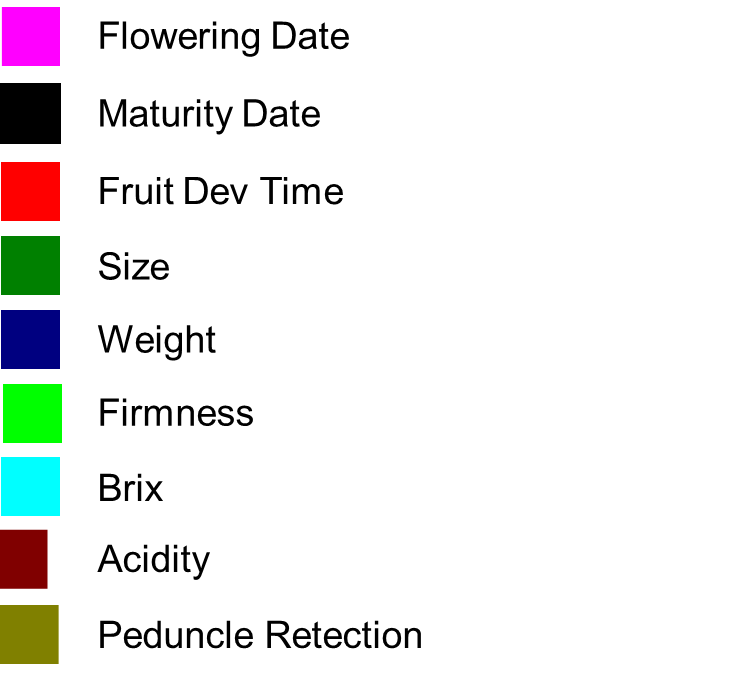

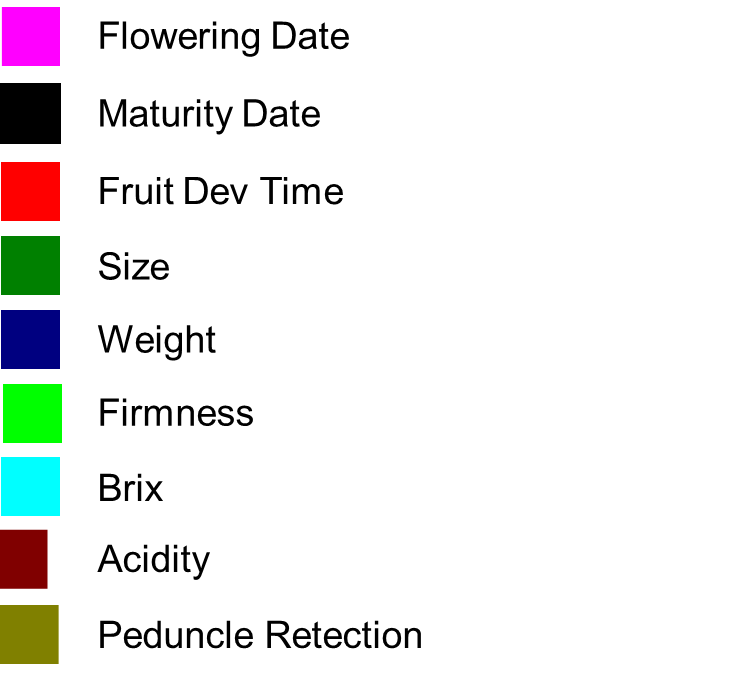


**Supplementary Fig 1** Genetic position of all QTLs detected both years in the consensus linkage map.
